# Spatial association between green space and COPD mortality: a township-level ecological study in Chongqing, China

**DOI:** 10.1186/s12890-023-02359-x

**Published:** 2023-03-17

**Authors:** Aiping Gou, Guanzheng Tan, Xianbin Ding, Jiangbo Wang, Yan Jiao, Chunyan Gou, Qiang Tan

**Affiliations:** 1 College of Ecological Technology and Engineering, Shanghai Institute of Technology, Shanghai, 201418 China; 2 Institute of Chronic and Non-communicable Disease Control and Prevention, Chongqing Center for Disease Control and Prevention, Chongqing, 400042 China; 3 College of Architecture, Nanjing Tech University, Nanjing, 211816 China; 4 Department of Acupuncture, Chongqing Traditional Chinese Medicine Hospital, Chongqing, 400021 China

**Keywords:** COPD, Green space, Chongqing, China, GWR, GAMs

## Abstract

**Background:**

There are regional differences in the effect of green space on mortality of Chronic obstructive pulmonary disease (COPD). We conduct an ecological study, using the administrative divisions of Chongqing townships in China as the basic unit, to investigate the association between COPD mortality and green space based on data of 313,013 COPD deaths in Chongqing from 2012 to 2020. Green space is defined by Fractional vegetation cover (FVC), which is further calculated based on the normalised vegetation index (NDVI) from satellite remote sensing imagery maps.

**Methods:**

After processing the data, the non-linear relationship between green space and COPD mortality is revealed by generalised additive models; the spatial differences between green space and COPD mortality is described by geographically weighted regression models; and finally, the interpretive power and interaction of each factor on the spatial distribution of COPD mortality is examined by a geographic probe.

**Results:**

The results show that the FVC local regression coefficients ranged from − 0.0397 to 0.0478, 63.0% of the regions in Chongqing have a positive correlation between green space and COPD mortality while 37.0% of the regions mainly in the northeast and west have a negative correlation. The interpretive power of the FVC factor on the spatial distribution of COPD mortality is 0.08.

**Conclusions:**

Green space may be a potential risk factor for increased COPD mortality in some regions of Chongqing. This study is the first to reveal the relationship between COPD mortality and green space in Chongqing at the township scale, providing a basis for public health policy formulation in Chongqing.

## Introduction

COPD is a common chronic disease with airflow obstruction and incomplete reversibility [1], and acute exacerbations of COPD increase the socio-economic burden [2]. It is a major cause of increased morbidity, mortality and health care costs for chronic diseases worldwide [3]. The prevalence of COPD in most regions of China is higher than that estimated by the World Health Organization Model [4], and the number of COPD-related deaths in China in 2013 was 910,809, accounting for 31.1% of all COPD deaths in the world [5]. In order to make effective public health policy on COPD, it is important to explore the risk factors for COPD deaths.

Green space is an integral part of the habitat, and studies have shown that people in urban areas with high green space coverage are at lower risk of chronic diseases, including cardiovascular disease [6], asthma [7] and diabetes [8]. Greater exposure to the natural environment can promote overall human health and well-being [9].

However, there are inconsistencies in the effects of green space on respiratory health. A study in the Netherlands found that increased green space would reduce the prevalence of COPD [10], and another study in the UK found similar findings [11]. However, in contrast to the two studies, a national cross-sectional study in China found that green space in community may be a risk factor for increased COPD prevalence, especially true in the northern and north-eastern China [12]. In addition, a Korean cross-sectional study and a Hong Kong cohort study found no significant association between green space and mortality from respiratory diseases [13, 14]. The inconsistency of these studies may imply that there is inter-regional variability in the effect of green space on COPD.

There is no concrete evidence for this inconsistency, but it is generally accepted that possible explanations include some subjective bias in the quantification of green space in some studies, or related to inter-regional differences in vegetation types and methods of COPD ascertainment [12]. On one hand, green space can reduce air pollution and thus reduce adverse respiratory effects [15], on the other hand, some plants may release volatile organic compounds (VOCs) [16]. A study in the USA found a positive correlation between asthma in children and levels of green space around their homes, attributed to VOCs [17].

It is an effective tool for future planning, health management and evaluation to clarify the factors contributing to the spatial pattern of disease. There are few studies on the relationship between the green space and the prevalence of COPD, and the influence of the green space on the COPD mortality may be different in different regions. In order to improve health management and control COPD mortality in Chongqing, we conducted an ecological study in Chongqing based on township administrative divisions as the basic unit, with the following objectives: (1) To investigate the spatial distribution of COPD mortality in Chongqing; (2) To reveal the influence of green space on COPD mortality in Chongqing and its regional differences.

## Methods

### Study area

This research is based on the administrative territorial entity of Chinese villages and towns, which is the fourth level of administrative division in China. The study area, Chongqing, is a municipality directly under the Central Government of China in southwest China, with a hilly and mountainous terrain and a population mainly concentrated in the nine districts of the main city of Chongqing. The total resident population is 32,124,000 (2022), covering an area of 82,400 square kilometers, including 38 districts and counties, with 1,031 township-level divisions [18].

### Data

*Health Data* COPD mortality data was collected from Chongqing Center for Disease Prevention and Control from all districts and counties, with a total of 313,013 COPD deaths from 2012 to 2020. The COPD mortality rate for each township was then obtained by field calculation in QGIS.

*Green Space* In this study, FVC was used to quantify the green space of each area. FVC quantifies the denseness of vegetation and reflects its growth status, which is an important fundamental data for describing ecosystems and has been widely used in various fields [19–21]. In this study, the Sentinel-2A satellite was selected by the Google Earth Engine platform to obtain remote sensing images with less than 20% cloud cover in Chongqing in 2020, and the NDVI data were obtained by de-clouding, stitching and calculating. The vegetation coverage was then retrieved from the NDVI data using a pixel-wise dichotomous model [22], which is expressed as:

1 $${f}_{vc}=\frac{NDVI-{NDVI}_{soil}}{{NDVI}_{veg}-{NDVI}_{soil}}$$

**Fig. 1:**
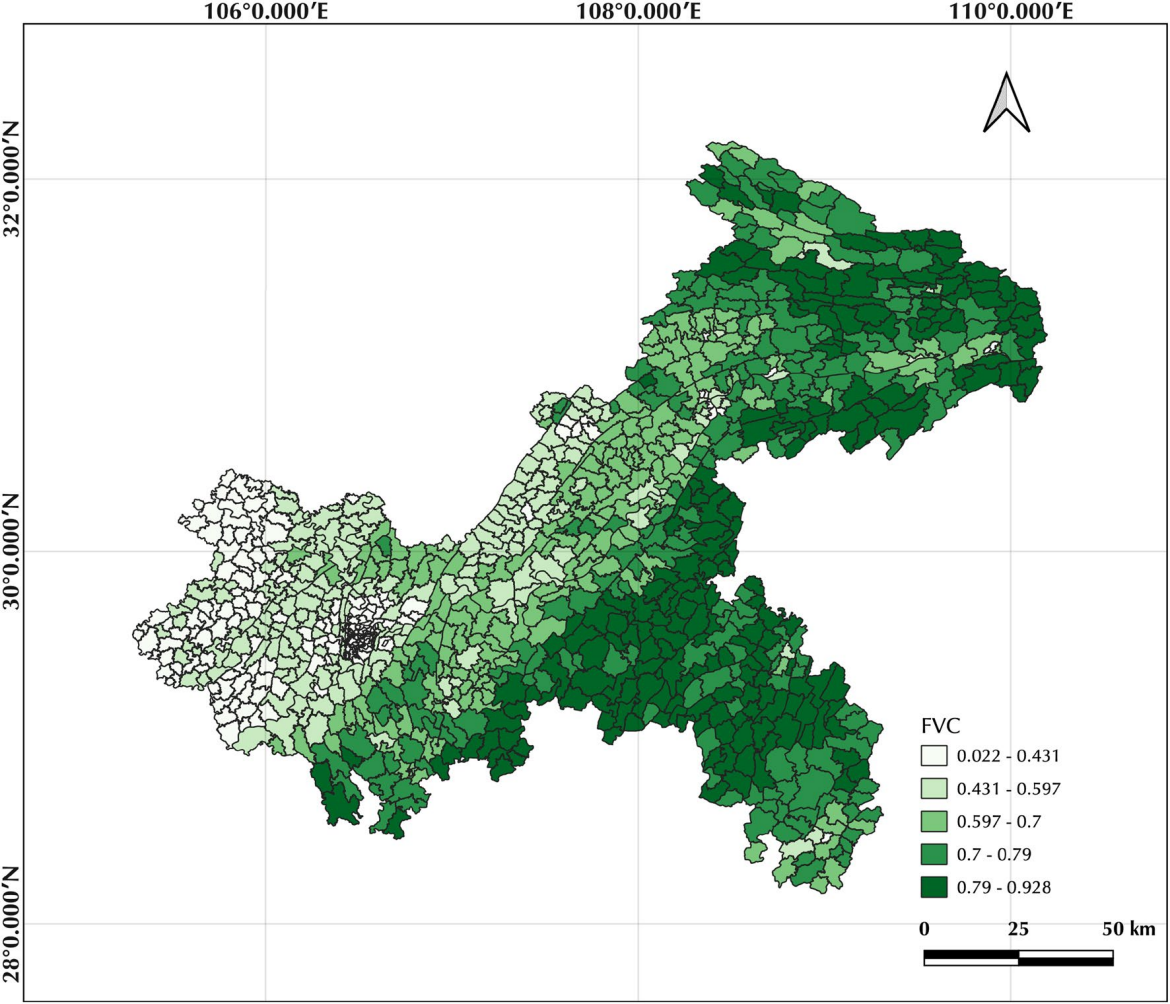
Mean values of FVC by quintile for each township

The ${NDVI}_{soil}$ is the minimum value of the pure soil image, and ${NDVI}_{veg}$ is the maximum value of the pure vegetation image. The values of the cumulative frequency of the NDVI image were selected as the values of ${NDVI}_{soil}$ and ${NDVI}_{veg}$ respectively based on the 5% and 95% confidence intervals in the study. The NDVI frequency histograms were calculated after the removal of the water areas in order to avoid the influence of the large water areas. The final FVC raster data was obtained at 10 m resolution. Based on the raster data, the mean FVC values for each township in Chongqing were calculated in QGIS (Fig. 1).

Other data: Population data for each township are based on the sixth census (2010), from the Chongqing Municipal Bureau of Statistics (2022), where the proportion of elderly population is calculated from the number of people over 65 years in each region and the proportion of gender is calculated by females. Air pollution is widely considered to be significantly associated with COPD mortality [23–25]. PM2.5 and PM10 data were obtained from a 1 km resolution daily raster data set produced by the State Key Laboratory of Remote Sensing Science, Beijing Normal University [26, 27], and the annual average raster data of PM2.5 and PM10 were calculated separately in this paper by the raster package of R.

The spatial distribution of medical resources also has an important impact on the spatial distribution of COPD mortality. In this paper, Point of Interest (POI) spatial distribution data of medical resources within Chongqing city of Gaode Map was obtained through python, and a total of 1777 POI points were obtained. In order to conduct further statistical analysis, the kernel density of these POI points was estimated by QGIS, where the influence radius was set to 10 km, and the corresponding weights were assigned according to the different levels of medical institutions of the POI points, and finally the raster of the density distribution of medical institutions within Chongqing was calculated.

### Statistical analysis

The article summarizes the characteristics of the data in descriptive statistics. At the same time, in order to explore the spatial relationship of COPD mortality, a spatial autocorrelation analysis of COPD mortality was performed, and its spatial correlation was expressed by the Moran index [28, 29]. Then, to explore the binary relationship between the variables, a spearman correlation analysis [30] was performed on each variable to obtain the binary correlation coefficient between the variables. Based on the results of the spearman correlation coefficients, variables with significant collinearity and non-significant correlation with COPD mortality were removed and finally three variables of FVC, proportion of elderly population, and density of health facilities, were selected for regression analysis.

Based on the results of the spearman correlation analysis, the study first attempted to describe the relationship between COPD mortality and FVC using GAMs Model, which is able to examine the relationship between the dependent variable and multiple independent variables as well as to fit the model through a non-linear smoothing term [31]. The general expression for GAMs is:

2 $$g(Y)=\alpha +\sum_{j=1}^{n} {f}_{i}\left({x}_{j}\right)+\varepsilon$$

where *Y* is the dependent variable, $g(Y)$ is the link function, $\alpha$ is the intercept term, ${x}_{j}$ is the independent variable, ${f}_{i}\left({x}_{j}\right)$ is the smoothing function, and $\varepsilon$ is the random error. Where the smoothing parameters will be calculated by the restricted maximum likelihood method of smoothing (REML) to ensure stable and reliable results [32]. The process of constructing the GAMs in this paper consists of three models with increasing adjustment levels, as follows:

3 $$\mathrm{Model} 1: g\left(Deaths\right)=\alpha +s\left(FVC\right)+\varepsilon$$

4 $$\mathrm{Model }2: g\left(Deaths\right)=\alpha +s\left(FVC\right)+s(Hospital)+\varepsilon$$

5 $$\mathrm{Model }3: g\left(Deaths\right)=\alpha +s\left(FVC\right)+s(Hospital)+s(Older)+\varepsilon$$

where $Deaths$ is the COPD mortality rate in each township, $FVC$ is the vegetation cover, $Hospital$ is the spatial distribution density of medical institutions, and $s()$ is the natural spline smoothing function.

Based on the results of the spatial autocorrelation analysis, GWR was performed on the independent and dependent variables in order to expose the spatial association between green space and COPD mortality. GWR is essentially an improved global regression model, where GWR fits a local regression equation at each spatial location, resulting in local regression coefficients that reflect the relationship between the independent and dependent variables for each township unit, as well as the spatial heterogeneity of each region [33, 34]. The general formula for the GWR model is:

6 $${y}_{i}={\beta }_{0}\left({u}_{i},{v}_{i}\right)+\sum_{k=1}^{t} {\beta }_{k}\left({u}_{i},{v}_{i}\right){x}_{ki}+{\varepsilon }_{i}$$

where ${u}_{i}$ is the latitude of the *i-th* location, ${v}_{i}$ is the longitude of the *i-th* location, ${\beta }_{0}$ is the regression constant for the *i-th* location, ${\beta }_{k}$ is the *k-th* regression parameter to be estimated for the *i-th* location, ${x}_{ki}$ is the observed value of the *k-th* variable for the *i-th* location, *t* is the number of independent variables, and ${\varepsilon }_{i}$ is the random error for *i-th* location. The spatial weight function of GWR affects the parameters such as local regression coefficients, and the Gauss function is used to determine the weights in this study. The value of bandwidth affects the analysis results of the model, and the method selected in this study is corrected Akaike Information Criterion (AICc) [35], which finally result in an optimal bandwidth of 18.

Finally, to further investigate the interaction between the spatial correlation intensity and variables of green space and COPD mortality, the Geo-detector was used for factor detection and interaction detection [36]. It is important to note that as the data used were continuous, the discrete transformation of spatial data into class variables was carried out before the detection of factors and interactions.

The construction of the GAMs model for this study was performed using the “mgcv” package in R (4.2.1), with the selection of spline curves relying on the “splines” package (R Core Team 2022); the GWR was constructed using the “GWmodel” package [37, 38]; the “GD” package was used for the Geo-detector [39]; and the spatial autocorrelation analysis was performed using GeoDa (1.10); the calculation and processing of raster data relied on the “raster” package [40], the “sf” package [41] and QGIS (3.26).

## Results

### Descriptive statistics and spearman correlation

**Fig. 2:**
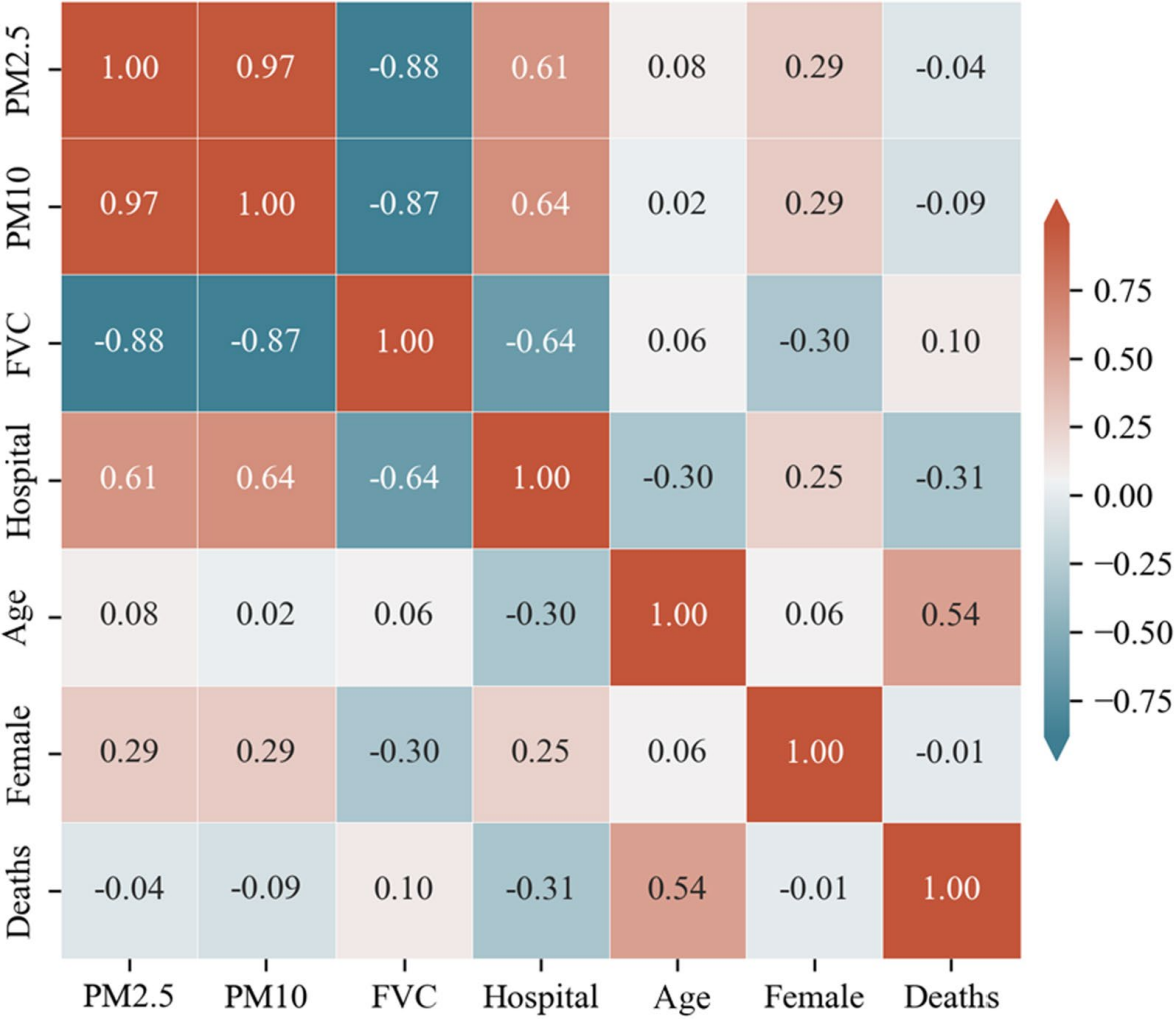
Bivariate correlation matrix graph. Deaths is the mortality rate between 2012 and 2020 for each communal administrative unit; FVC is the fractional vegetation cover; Hospital is the nuclear density value of medical institutions in each region; Age is the percentage of population over 65 years old in each region; Female is the percentage of female population in each region. Red cells indicate a positive correlation, Blue cells a negative correlation

**Fig. 3:**
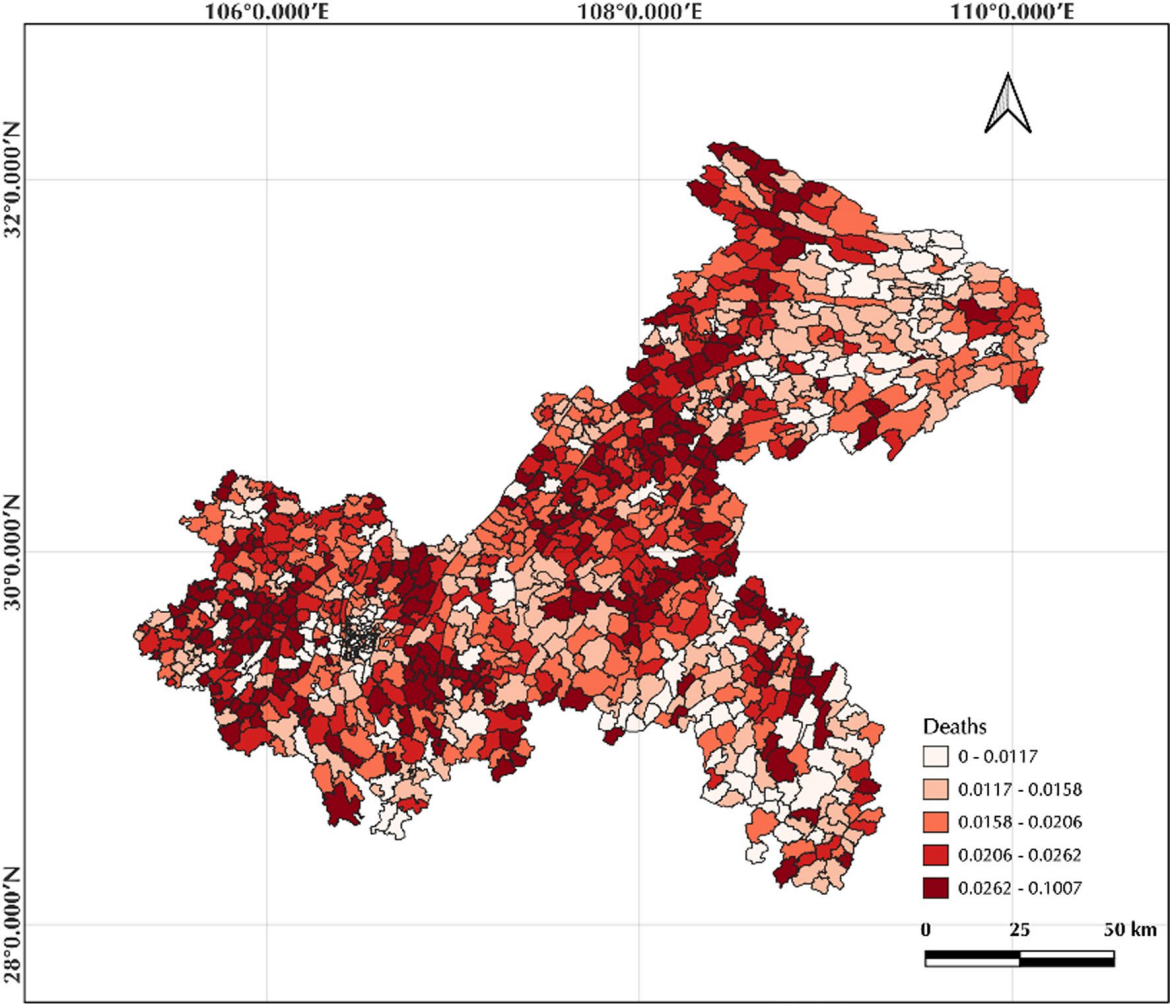
COPD mortality by quintile

The descriptive statistics for all variables are shown in Table 1. The multi-year mortality rates for each township administrative unit of COPD in Chongqing from 2012 to 2020 range from 0 to 0.1, with a mean of 0.0191 and a standard deviation standard deviation (SD) of 0.009. The Spearman correlation coefficient is able to describe the correlation between the variables. It takes values in the range − 1 to 1. A positive value means that the two variables are positively correlated, while a negative value means the opposite [30]. Figure 2 shows the Spearman correlation between all variables, with a correlation of 0.1 between COPD mortality and FVC (*p* < 0.01), and a significant correlation between FVC and PM2.5, PM10 (|correlation coefficient|> 0.85, *p* < 0.01), these variables were removed to avoid Multicollinearity effects. Figure 3 shows the COPD mortality rates by township administrative unit in Chongqing, with values ranging from 0 to 0.1007, with high values mainly in the central, northern and western regions of Chongqing around the municipality. The values of FVC range from 0.0223, to 0.928, with high values mainly in the southeast and northwest regions.

**Table 1:** Descriptive statistics

	Min.	Max.	Median	Mean	SD
Deaths	0	0.1007	0.018	0.0191	0.009
FVC	0.0223	0.9280	0.6552	0.6091	0.1946
Hospital	0	955.701	3.6222	54.600	153.8525
Age	0.0046	0.8499	0.1335	0.1379	0.0681
PM2.5	20.36	35.38	29.14	28.37	3.772
PM10	33.54	55.91	45.69	44.56	5.8480
Female	0.4075	0.5693	0.4905	0.4898	0.01439

Deaths is the mortality rate between 2012 and 2020 for each communal administrative unit; FVC is the fractional vegetation cover; Hospital is the nuclear density value of medical institutions in each region; Age is the percentage of population over 65 years old in each region; Female is the percentage of female population in each region.

### Spatial autocorrelation of COPD mortality

**Fig. 4:**
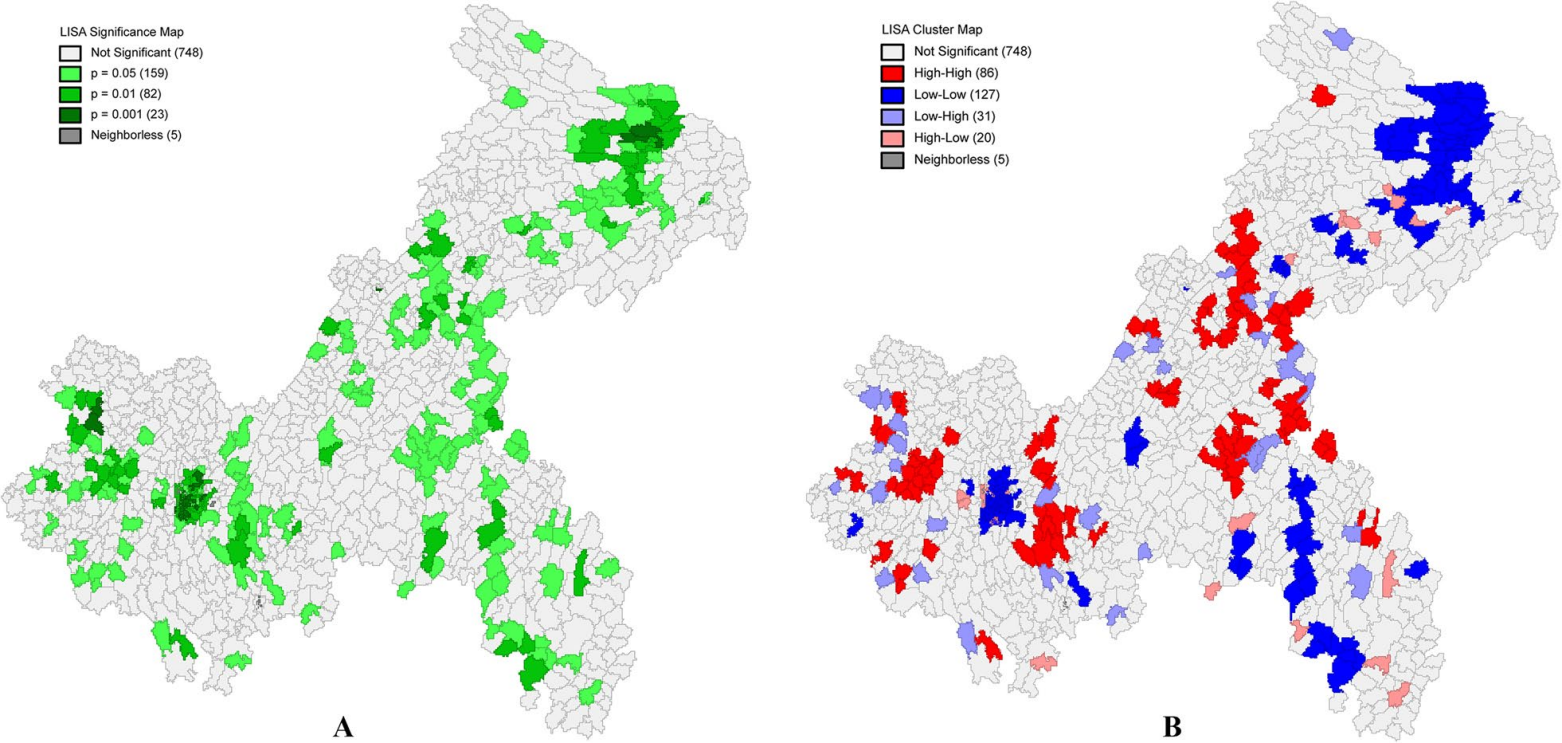
**A** is the Significant level; **B** is Spatial autocorrelation

To test whether COPD mortality is spatially autocorrelated, the study used Moran's I spatial autocorrelation analysis. The Moran's I index for COPD mortality was 0.257 (*p* < 0.01), implying a potential dependence on the spatial distribution of COPD mortality in Chongqing. The LISA Cluster Map (Fig. 4) also shows the spatial distribution of the clusters and the corresponding confidence of each region; the high value clusters are located in the central and western part of the main urban area; while the low value clusters are distributed in the north-eastern, south-eastern and western areas of the main city.

### Generalised additive models results

According to the variance inflation factor (VIF) test, the VIF of each variable is less than 5, and there is no multicollinearity between the variables [42]. Table 2 shows the results of the GAMs regression, where degrees of freedom greater than 1 means a non-linearly relationship between the explanatory variable and the COPD mortality; the F statistic reflects the degree of influence of the explanatory variables, the larger the F value the greater the influence; the P value is a parameter to judge the hypothesis test; and the R^2^ reflects the effect of the model fit. Model 1, Model 2 and Model 3 are three models with increasing adjustment levels respectively. Model 1 is a univariate regression model describing the relationship between FVC and COPD mortality; In Model 2, R^2^ was 0.183 after adding the medical resource covariates, and the rate of deviation explanation was 21.5%; In Model 3, the proportion of elderly population was also added and the coefficients of all variables are statistically significant, and the final model deviation interpretation rate is 32.4%.

**Table 2:** Parameters of GAMs results

	Mode1 (R^2^ = 0.074)				Model 2 (R^2^ = 0.183)				Model 3 (R^2^ = 0.287)			
Variable	edf	Ref.df	F	*P*	edf	Ref.df	F	*P*	edf	Ref.df	F	*P*
FVC	6.803	7.918	9.962	0	6.260	7.439	7.107	0	5.3	6.591	2.276	0.02
Hospital					5.664	6.792	19.957	0	2.861	3.558	3.536	0.01
Age									3.586	4.270	42.918	0

*Edf* Estimated degree of freedom, *Ref.df* Reference degree of freedom

### Geographically weighted regression models results

**Fig. 5:**
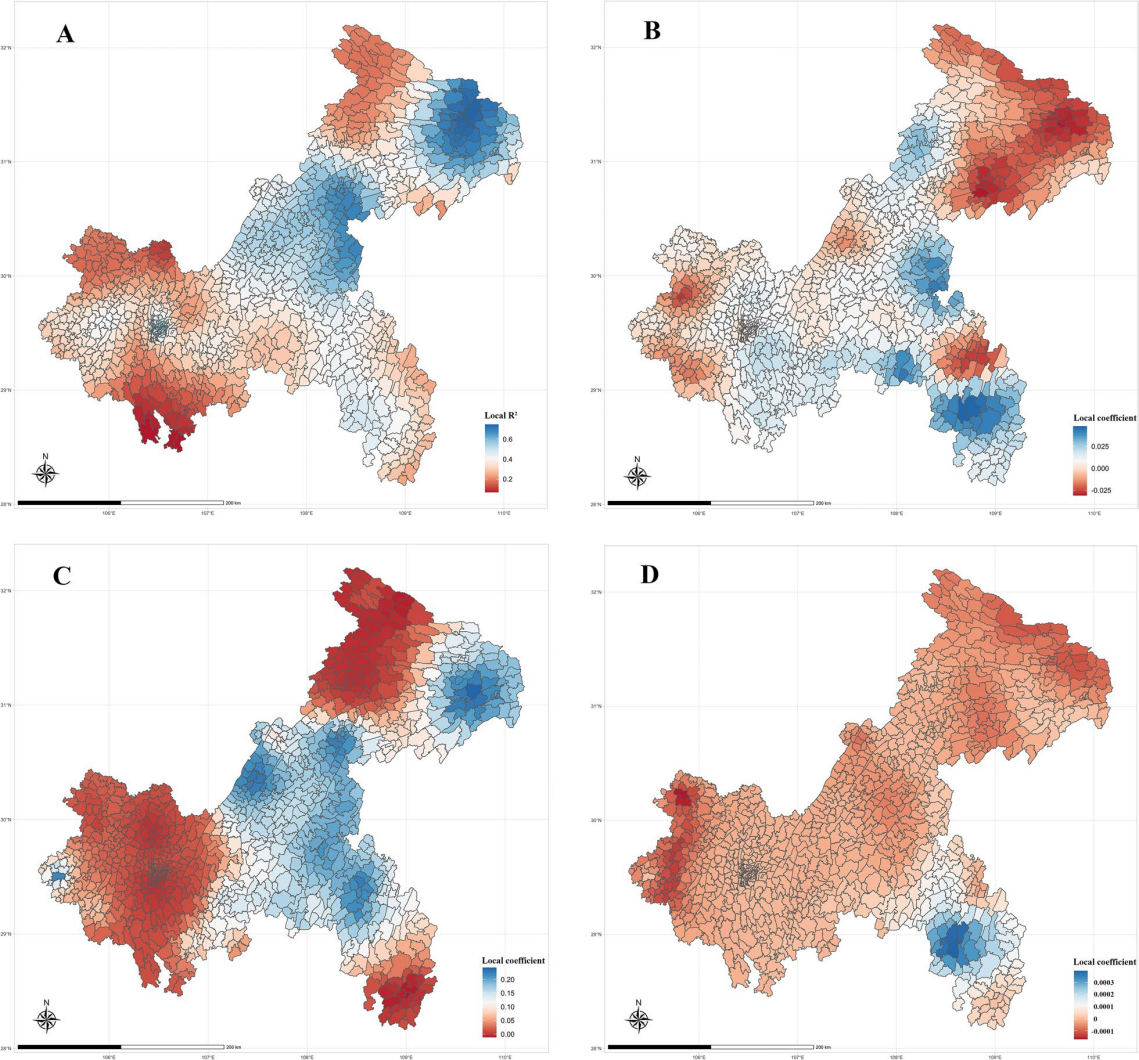
**A** is the R^2^ of the geographically weighted regression model; **B** is the coefficient of the variable FVC; **C** is the coefficient of the variable Age; **D** is the coefficient of the variable Hospital

The relationship between COPD mortality and FVC was spatially unstable as shown in Fig. 5. From the results of regional parameter (Table 3), the FVC local regression coefficients range from − 0.0397 to 0.0478, 63.0% of the regions are positive and 37.0% are negative. The negative correlation areas are mainly located in the northeast and west of Chongqing. The local regression coefficients for Hospital range from − 0.0001 to 0.0004, a smaller range than that of the other two variables, with 19.4% of the area being positive and 81.6% being negative, the positive impact area is distributed in the southeast region. The Age local regression coefficients range from − 0.0161 to 0.2428, 91.7% of the areas are positive and 8.3% are negative. The high-value regions are mainly in the central and northeastern parts of Chongqing.

**Table 3:** Parameters of GWR results

Variable	Min	Median	Max	*P* value	+ (%)	− (%)
Intercept	− 0.0375	0.0096	0.0362	< 0.001	65.3	34.7
FVC	− 0.0397	0.0048	0.0478	< 0.001	63.0	37.0
Hospital	− 0.0001	− 0.00001	0.0004	< 0.001	19.4	81.6
Age	− 0.0161	0.0664	0.2428	< 0.001	91.7	8.3

+ Percentage of areas where the sign of the coefficient is a positive sign

− Percentage of areas where the sign of the coefficient is a negative sign

### Geo-detector results

According to the Q-value (*P* value < 0.01) of Geo-detector factor detection, the three variables selected to explain the change in COPD mortality in Chongqing are Age, Hospital and FVC in descending order of explanatory power, with 0.257, 0.142 and 0.08 respectively. The difference in interpretive power when two factors work together on a single factor can be obtained by interactive probing. Among them, the interpretive power of FVC interaction with Age was 0.2885; the interpretive power of the interaction between FVC and Hospital was 0.2039; and the interpretive power of the interaction between Age and Hospital was 0.2846. All the results showed a two-factor enhancement, indicating that the interaction of factors had different degree of enhancement in explaining the mortality of COPD compared with single factor.

## Discussion

### Interpretation of results

This study found that the spatial distribution of COPD mortality in Chongqing has certain aggregation characteristics. The non-linear relationship between FVC and COPD mortality in Chongqing was described by GAMS model, and the model was made more stable by adding covariates. It was found that COPD mortality changed in segments as the FVC value increased. Based on the results of the GAMs, we hypothesized that there were regional differences in the association between COPD mortality and FVC, and the results of the GWR verified this hypothesis (Fig. 5). COPD mortality was positively correlated with FVC in 63% of the township areas in Chongqing and negatively correlated in 37.0% of the areas. It means that COPD mortality increases with increasing FVC in 63% of the township areas in Chongqing, higher vegetation cover brings higher risk of COPD mortality, COPD mortality decreases with increasing FVC in 37% of the township areas, and the negatively correlated areas are mainly located in the northeast of Chongqing. And according to the results of R^2^ distribution, the GWR model effect was better in this region. Overall, there was a spatially non-stationary relationship between COPD mortality and FVC in Chongqing.

Considering that regional differences in air pollution may affect the judgment of the relationship between FVC and COPD mortality, the study revealed the relationship between FVC and PM2.5, PM10 through Spearman correlation analysis, with significant negative correlation coefficients of − 0.88 and − 0.87, respectively. Implying that in Chongqing, the areas with high FVC have lower PM2.5 and PM10 concentrations. Therefore this study was able to rule out the possibility that high PM2.5 and PM10 concentrations would mask the beneficial effect of FVC on COPD mortality, making the findings more convincing. Also according to the results of the GAMs, GWR and Geo-detector models, the age factor makes the largest contribution to the distribution of COPD mortality, and the correlation coefficients for FVC are all relatively small. Such results are also in line with the reality.

Regional differences in the relationship between green space and COPD mortality is acceptable. A Belgian study including mortality data from five urban areas between 2001 and 2011 found a negative association between residential green space and respiratory disease mortality [43]; a Korean cross-sectional study and a Chinese cohort study found no significant association between green space and respiratory disease mortality [13, 14]; a national study of China by Fan [12] concluded that green space was a risk factor for increased COPD prevalence, and according to similar studies, findings on the relationship between green space and respiratory disease vary widely across regions.

In most townships in Chongqing where COPD mortality is positively associated with FVC, one possible explanation for the predominance is that areas with high FVC tend to be rural and mountainous, with limited access to medical resources, resulting in high mortality and high FVC occurring in the same areas. From Fig. 3, it can be seen that the mortality rate of COPD in the west of Chongqing is lower and the medical resource there is the best, while the townships surrounding the main urban area have correspondingly higher FVC values, but also lower availability of medical resources and higher COPD mortality rates. Another possible explanation is that some high-value FVC regions have lower urbanization with industrial structure dominated by primary and secondary industries, with high poverty and unemployment rates, low health insurance coverage, and a high proportion of the population currently or previously engaged in manual work. Studies have shown that people with low incomes are more susceptible to air pollution [13, 44], and people with low levels of education may also lack knowledge of air pollution disease prevention [45], resulting in higher COPD mortality. The regions where COPD mortality is negatively correlated with FVC in Chongqing are mainly located in the northeast. The possible reasons are that these regions have excellent natural environment and some districts and towns are all-area tourism demonstration areas, dominated by tertiary industries. The green space forms a complete natural barrier to optimize air quality and block air pollution particles, thus providing a good environment and reducing the risk of respiratory diseases [15].

There were significant regional differences in the spatial correlation between COPD mortality and green space in Chongqing. The reason for this may be related to the degree of influence of the FVC factor on the distribution of COPD mortality. According to the results of the geographic probe, the interpretive power of FVC on the spatial subdivision of COPD mortality is only 0.08, which is inferior to age and hospital availability; and according to the factor interaction detection, the interpretive power of multi-factor interaction is greater than that of FVC single factor, implying that the spatial subdivision of COPD mortality should be explained by multiple factors.

### Limitations

Although the results of this study are statically significant, there are some limitations. The results of this study may be confounded by some difficult-to-measure personal factors due to the use of the township as the basic study unit, where many data were missing and only a few covariates could be taken into account. Firstly, smoking is considered to be a major cause of COPD, but the results of this study cannot eliminate the possible impact of smoking habits in the population due to the lack of data. Secondly, increased mortality of COPD may also be related to the presence of underlying disease in the cases themselves, and the presence of underlying disease in the study subjects themselves may have an impact on the findings. Meanwhile, due to data limitations, there were differences between the dependent variable COPD mortality (2012–2020) and the independent variable FVC (2020), so the study could not exclude uncertainties stemming from temporal inconsistencies [46].

## Conclusion

The control of COPD mortality is of great importance to alleviate social stress and improve people's health and well-being. Due to the correlation between green space and respiratory diseases, we attempted to uncover the spatial relationship between green space and COPD mortality in Chongqing. It is expected to achieve the control of COPD mortality through planning policy. However, the results did not confirm this expectation. Green space may be a potential risk factor for increased COPD mortality in some regions of Chongqing. Therefore, this spatial differentiation needs to be taken into account in future green space planning and public health policy development in Chongqing.

## Data Availability

The data of PM2.5 and PM10 can get from [ChinaHighPM2.5: Big Data Seamless 1 km Ground-level PM2.5 Dataset for China] at [https://doi.org/10.5281/zenodo.6398971] and [ChinaHighPM10: Big Data Seamless 1 km Ground-level PM10 Dataset for China] at [https://doi.org/10.5281/zenodo.6449937]. The population data can get from [Chongqing Bureau of Statistics] at [http://tjj.cq.gov.cn/wap.html]. Green space data can be calculated from the Google Earth Engine platform. But the COPD mortality data will be made available from the corresponding author on reasonable request due to privacy and ethical restrictions.
